# Using T4 genetics and Laemmli’s development of high-resolution SDS gel electrophoresis to reveal structural protein interactions controlling protein folding and phage self-assembly

**DOI:** 10.1016/j.jbc.2022.102463

**Published:** 2022-09-05

**Authors:** Jonathan King

**Affiliations:** Department of Biology, MIT, Cambridge, Massachusetts, USA

**Keywords:** SDS, acrylamide gels, electrophoresis, phage assembly, protein folding, phage genetics

## Abstract

One of the most transformative experimental techniques in the rise of modern molecular biology and biochemistry was the development of high-resolution sodium dodecyl sulfate polyacrylamide gel electrophoresis, which allowed separation of proteins—including structural proteins—in complex mixtures according to their molecular weights. Its development was intimately tied to investigations of the control of virus assembly within phage-infected cells. The method was developed by Ulrich K. Laemmli working in the virus structural group led by Aaron Klug at the famed Medical Research Council Laboratory for Molecular Biology at Cambridge, UK. While Laemmli was tackling T4 head assembly, I sat at the next bench working on T4 tail assembly. To date, Laemmli’s original paper has been cited almost 300,000 times. His gel procedure and our cooperation allowed us to sort out the sequential protein–protein interactions controlling the viral self-assembly pathways. It is still not fully appreciated that this control involved protein conformational change induced by interaction with an edge of the growing structure. Subsequent efforts of my students and I to understand how temperature-sensitive mutations interfered with assembly were important in revealing the intracellular off-pathway aggregation processes competing with productive protein folding. These misfolding processes slowed the initial productivity of the biotechnology industry. The article below describes the scientific origin, context, and sociology that supported these advances in protein biochemistry, protein expression, and virus assembly. The cooperation and collaboration that was integral to both the Laboratory for Molecular Biology culture and phage genetics fields were key to these endeavors.

One of the most widely used and important techniques in modern biology is SDS polyacrylamide gel electrophoresis. This technique was developed in 1970 by Ulrich K. Laemmli when he was a postdoctoral fellow with Aaron Klug in the Medical Research Council’s Laboratory of Molecular Biology (MRC LMB) in Cambridge, UK (1). Two hundred ninety thousand subsequent papers cite Laemmli’s original publication. This Reflections article provides an account of the conditions and scientific interactions that led to this invaluable technical development. Laemmli’s method, combined with the development of T4 conditional lethal mutants, allowed both of us to identify the phage structural proteins and structural protein interactions critical for T4 assembly. These experiments opened up valuable approaches to the general problem of capsid assembly in double-stranded DNA viruses, as well as to deeper understanding of the folding and misfolding of complex proteins.

Aaron Klug had emigrated to the UK from South Africa, coming from the progressive community that rejected apartheid. He joined Rosalind Franklin’s group in London to study nucleic acid structure and always championed her contribution to the revealing the double-stranded helical structure of DNA. Moving to the MRC LMB, Klug focused on virus structure and advancing electron microscope methodology.

Jacob V. Maizel Jr, who had made important contributions to the use of SDS and acrylamide gel analysis of poliovirus proteins, was visiting on sabbatical (2, 3). Aaron Klug had carried out an influential study of the nucleation and assembly of rod-shaped tobacco mosaic virus (4) and was extending his structural studies to spherical viruses. Laemmli, Maizel, and I—and later Sidney Altman—had come to the MRC LMB to work under Klug on the assembly of phage T4 and other spherical viruses.

Klug, with John Finch and Tony Crowther, was leading the effort to understand the structure of spherical viruses through electron microscopy and image processing. Laemmli and I were both postdoctoral fellows at the LMB working to develop electron microscopy and image processing skills. We were particularly welcome because improving these procedures required very uniform stable high-quality samples, which preserved their molecular organization in heavy metal stains. Our purified T4 phage capsids and tail components provided such samples. Crick, Brenner, and coworkers at the LMB had used T4 rII mutants to identify the triplet nature of the genetic code, and the lab had sterile glassware, media, and full support for T4 infection of *E. coli*.

Laemmli’s scientific training in Kellenberger’s group in Geneva involved both sophisticated phage genetics and physical biochemistry. My Caltech background similarly included both classical and microbial genetics and physical biochemistry. The latter was driven by the relationship with the Chemistry Dept, where Linus Pauling did his Nobel Prize work, and which also included Norman Davidson and Jerome Vinograd developing analytical ultracentrifugation.

## The need for separating structural proteins and origins of the Laemmli gels

Laemmli’s motivation in developing high-resolution SDS gels was to analyze the structural proteins of the capsid of phage T4. These proteins are strongly, though noncovalently, bonded and not soluble in aqueous buffers. In the 1960s, the groups of R. H. Epstein and Edward Kellenberger in Geneva and R. S. Edgar at Caltech had developed two classes of conditional lethal mutants of phage T4 which interfered with the assembly of the phage structural proteins. Conditional lethal mutants could be isolated in any and every gene that encoded an essential function (5, 6, 7). At that time, mutants of structural proteins were very rare, whether in yeast, Neurospora, flies, mice, or humans.

There were two classes of conditional lethals: temperature-sensitive (ts) mutants developed by Edgar (5) and nonsense or amber mutants developed by Epstein (6). In the latter case, the mutation generated a stop codon within the normal amino acid coding region of a gene of interest. Propagating these phage strains required the use of *E. coli* hosts with a mutant suppressor tRNA, which occasionally inserted an amino acid at a stop codon. In the wildtype nonpermissive host bacteria, the nonsense mutation resulted in premature termination of the polypeptide chain and translation of a smaller nonfunctional amber fragment. Isolation of the mutants depended on their growth on one host but not on the other.

For ts mutations, isolation was of phage strains that propagated at 25 °C or 30 °C but not at 39 °C to 40 °C. The development of these mutants affecting genes for structural proteins was reported in a seminal—if obscurely titled—paper published in the Cold Spring Harbor Symposia on Quantitative Biology (7).

Infection of cells with nonsense or ts mutations in genes for structural proteins often led to accumulation of morphogenetic intermediates in virus assembly. Laemmli, Kellenberger, and their coworkers in Geneva had characterized the capsid-related structures accumulating in cells infected with mutants defective in head assembly ((8)). As a graduate student at Caltech and working with Bob Edgar and later Bill Wood, I had identified the intermediates in tail assembly and tail fiber assembly (9, 10).

Discontinuous polyacrylamide gel electrophoresis had been invented by Baruch Davis and Leonard J. Ornstein working at New York’s Mt. Sinai hospital, in order to resolve the proteins in blood and related samples. They described their work in two important and classic papers published in the Annals of the New York Academy of Sciences (11, 12) that are still very much worth reading. The discontinuous gel systems of Ornstein depended on stacking and “compression” of protein species according to their isoelectric point and charge density between two different buffer systems. Ornstein’s papers explained how generating a sharp voltage gradient between the leading edge of one buffer and the trailing edge of a second buffer resulted in proteins of different charge forming narrow bands or discs, driven by the voltage gradient at the discontinuity. The tendency of the bands to spread was opposed by the need for a continuous voltage gradient. In these separations, proteins remained native, and their surface charge of their native states determined their behavior at the buffer interface. Once the proteins passed the discontinuity and entered the separating gel, they were fractionated according to charge and molecular weight.

Davis described systematic experiments searching for an optimal matrix and the reasons for settling on polyacrylamide: it was (a) transparent, (b) biologically unreactive, (c) chemically inert, (d) uncharged, and had (e) controllable pore size and (f) mechanical strength. He also provided detailed instructions for preparing gels reproducibly and reliably in the first of the pair of articles.

Though Laemmli could isolate capsid structures from phage-infected cells, he was unable to determine their protein composition, since they did not dissociate under native conditions. Jake Maizel had shown for polioviruses that the particles could be dissociated and solubilized through the use of the detergent SDS, including SDS in gels during fractionation. This work had led to their key discovery that in the presence of SDS, polypeptide chains migrated through acrylamide gels proportional to their molecular weight (3, 13). Maizel understood that this represented the unfolding of the polypeptide chain and coating with one SDS molecule per peptide bond, generating an elongated rod-shaped complex in which size was proportional to chain molecular weight. Unfortunately, in these early SDS gels, the SDS/polypeptide chain complexes migrated as broad bands. This was adequate for poliovirus with only four protein components (14). However, for T4, with dozens of proteins needed for particle assembly, the resolution was inadequate.

Laemmli had been educated in the Swiss technical system and had a much deeper knowledge of electrochemistry than most of our peer molecular biologists, virologists, and geneticists. He recognized that it should be possible to get the stacking phenomena to work for an SDS polypeptide chain complex and therefore theoretically obtain high resolution under denaturing conditions. He set about trying to find a discontinuous buffer system in which the SDS/polypeptide chains would concentrate and stack at a buffer interface in the stacking gel above the separating gel. This involved making up many buffer and gel solutions, casting gels in glass tubes, running samples, then cracking open the glass tubes, slicing, drying, and staining the gel slices.

Since my work on T4 tail assembly was also stymied by the inability to resolve the more than 20 proteins involved (9), I recognized the value of his goal and happily provided an additional pair of hands to explore the many buffer and gel concentration variations that led to the final published method. Laemmli was an intense and hard worker, and my memories are of breathing SDS aerosols which we sprayed on the gel tops to get flat menisci and regular exposure to acrylamide through handling the gels for the slicing and drying, prior to staining. I’m not sure at what point we learned that acrylamide was a neurotoxin that could be absorbed through the skin and that breathing aerosols of SDS was not the best treatment of one’s respiratory tract. Though we did not smoke in the midst of experiments, after a day’s work, we would sit down for a cigarette. (Decades later I came down with cancer of the larynx, which I attributed to the holes punched in my vocal cord membranes by the SDS detergent aerosols, which then provided access of cigarette smoke and particles to the epithelial cells. This is a mechanism thought to account for the increased cancer of the larynx in smokers who are also heavy drinkers).

## Elucidation of major steps in dsDNA capsid assembly

Laemmli succeeded in finding a pair of buffers in which the SDS/polypeptide chains stacked at the discontinuity. Using this gel system, he was able to show that T4 heads were assembled from more than six different proteins and identify them as the products of specific T4 genes (1). Since the phenotype of mutant-infected cells as visualized in the electron microscope had already been determined, he was able to define the pathway of T4 head morphogenesis (8). One striking feature was the presence of defined proteolytic cleavages of a number of the structural proteins, within the organized lattice, coupled to the stages of icosahedral lattice transformation. These cleavages were clearly important in controlling capsid assembly, but they did make subsequent analysis more difficult.

**Figure 1:**
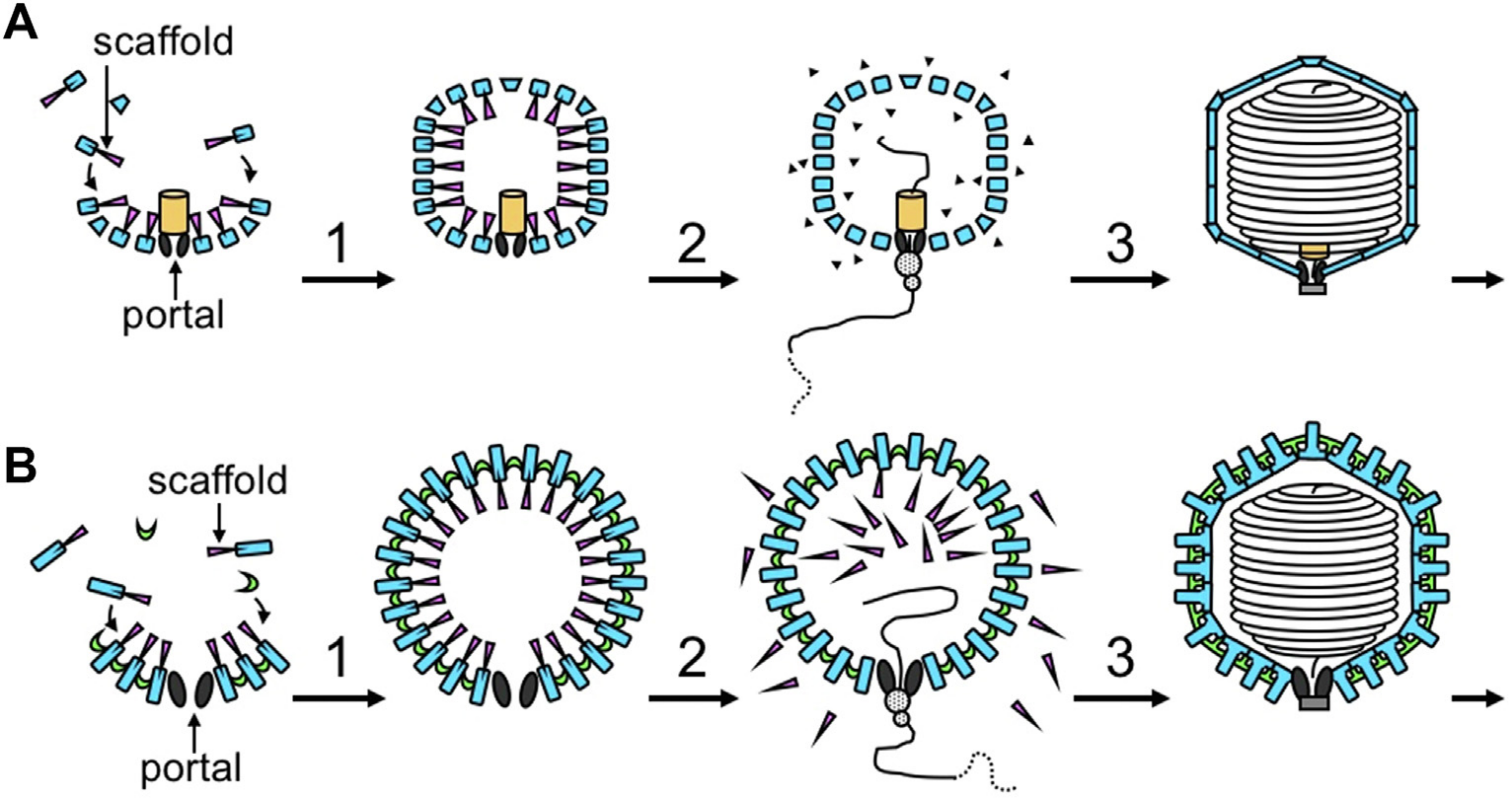
**General pathways for the assembly and DNA packaging in dsDNA phages**. For the well-studied dsDNA phages, a precursor shell is assembled empty of DNA, and the DNA is actively pumped into the structure through the unique portal vertex. This vertex also initiates procapsid assembly. “*A*” shows the T4-like pathway, in which the scaffolding protein is proteolyzed and is thus absent from the mature virus. “*B*” shows the P22-like pathway, in which scaffolding subunits exit through the lattice of the procapsids and are thus absent from the native state. The P22 gene 8 scaffolding subunits recycle for further rounds of assembly (17). In the absence of scaffolding subunits the coat subunits fail to form closed shells.

One of the essential proteins, the product of gene 22, was completely proteolyzed and was absent in the mature virion. This was subsequently shown to be the major scaffolding protein for T4 head assembly (15, 16). This scaffolding function—required for subunit assembly but removed prior to DNA packaging—was clarified when Sherwood Casjens, David Botstein, and I showed that in the Phage P22, the scaffolding exited and recycled, without proteolysis (17). These experiments made clear the very surprising fact that a precursor shell is first assembled and then the DNA pumped in through a unique portal vertex (18, 19). Over the following 4 decades, analysis of adenovirus, herpesviruses, and cyanophages revealed that all dsDNA viruses use this general pathway. Such pathways are illustrated in Fig. 1.

Laemmli and I immediately used his gel system to identify the T4 proteins needed for tail and tail fiber assembly (20, 21). Our 1971 paper had a more detailed description of the buffers and procedures in its Materials and Methods and was helpful to many investigators.

An unexpected result at the time was that all the phage structural proteins were translated at the same rate, regardless of whether or not they were assembled into phage structures. Feedback loops between assembly and transcription and translation were minimal, contrary to the models of gene expression from phage lambda and repressor proteins.

The tail fibers also required a phage-specified chaperone, the gene 57 product, and the long T4 fiber proteins failed to fold properly in its absence and therefore accumulated in the pellet as an inclusion body. Laemmli went on to demonstrate that the folding of the major gp23 coat protein required a nonstructural protein gp31, which we now know to be phage-specific replacement of the groES subunit of the GroEL/S chaperonin (22).

## Sequential assembly of the long tail fibers of T4

In the very first micrographs of T4, one of the distinctive morphological features were the long 1600 Å tail fibers which functioned in the initial recognition of and binding to host cells. The distal tip of the fibers recognize lipopolysaccharide receptors on the *E. coli* surface. Binding to cell surface is accompanied a major conformational change in the phage baseplate, triggering contraction of the sheath and penetration of the tail tube tip through the bacterial cell wall and membrane.

**Figure 2:**
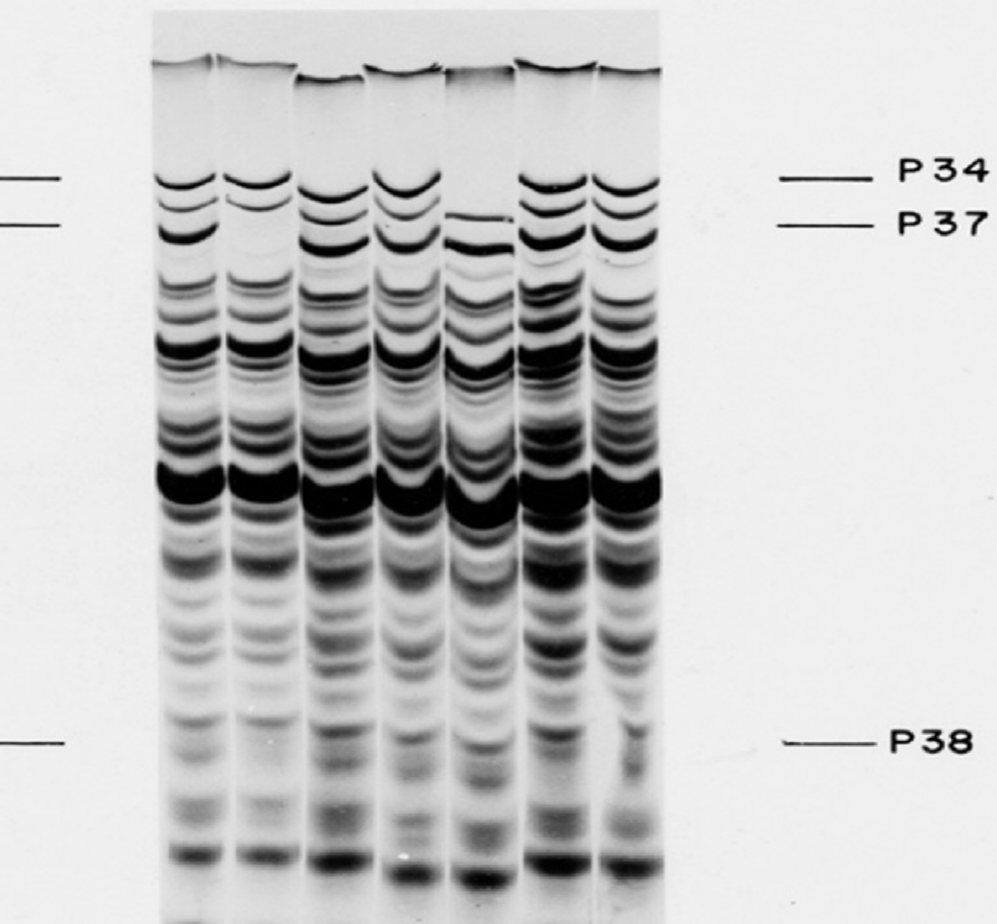
**Autoradiogram of**^**14**^**C-amino acid–labeled polypeptide chains synthesized in wildtype and mutant T4 infected *E. coli* cells**. The samples have been heated to boiling prior to electrophoresis so that all bands represent SDS/polypeptide chain complexes, which are separated according to molecular weight (3). The left lane are the proteins from wildtype infection. The other lanes show the lysates of cells infected with phage carrying an amber mutation in one of the genes needed for tail fiber assembly. Single missing bands identify the products of the mutant phage genes in those mutant-infected cells—P34, P37, and P38 (20). The proteins are named for their genes.

Edgar and Lielausis (1965) showed that a cluster of five genes (34, 35, 36, 37, and 38) were required for the assembly of the fibers and that rabbit antibodies neutralizing T4 bound to these fibers. One of the first uses of Laemmli’s gel system was identifying the protein products of the tail fiber genes (20). Phage proteins were labeled by uptake of radioactive amino acids to enhance the sensitivity of the SDS gel analysis. The 34 and 37 genes encoded very long polypeptide chains of 150,000 and 125,000 Da, respectively. The gel patterns of the proteins in these lysates highlighting the missing proteins from mutant lysates are shown in Fig. 2. Using sucrose gradient centrifugation and assaying the fractions with specific antibodies (13), I found that each of these very large proteins formed one of the half tail fibers (10) Both the distal and proximal half fibers are slender trimers of intimately interacting extended chains, in some regions triple coiled-coil–like structures and in others closer to triple beta helices (23, 24).

**Figure 3:**
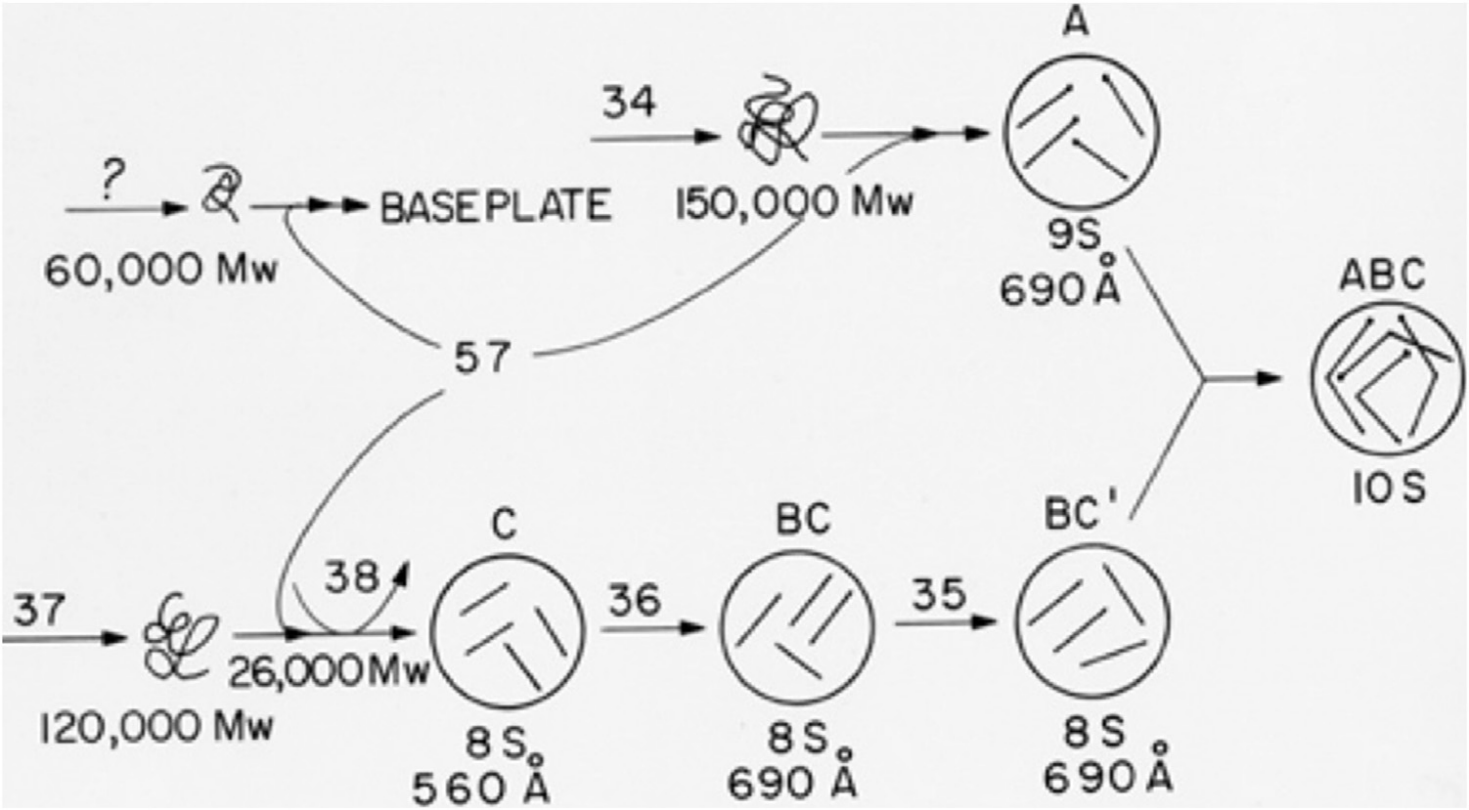
**Pathway for the assembly of the long tail fibers of Bacteriophage T4**. Proteins are numbered for the genes that encode them. The *A*, *B*, and *C* labels represent antigenic activities of the fiber proteins defined by rabbit antiphage antibodies (13). Note that the proximal p34 “A” half fiber only binds to the phage baseplate after it has joined with the “BC” distal fiber, through the small joint proteins p35 and p36. Presumably some conformational signal is propagated from the elbow joint to the terminus of the proximal half fiber, activating it for baseplate binding. In fact that step is even more complex, and uses a catalytic protein, the product of gene 63, for efficient binding (48). The tip of the distal fiber that recognizes the host receptor, formed of the gp38 protein, has been crystallized and is a complex triple B-strand fold with multiple iron atoms shown in Fig. 4 (23). (Bartual, Ogero, Garcea-Doval and van Raaij, 2010).

Using the rabbit-specific antibodies, we found that the smaller proteins encoded by genes 35 and 36 together form the binding site and elbow joint between the proximal and distal half fibers. Most striking was that the proximal half fiber did not bind to the phage baseplate, not until it was assembled with the proximal half fiber into the complete fiber. This was direct evidence that different parts of the phage were prefabricated before they could interact to form a functional structure. The steps in the tail fiber assembly pathway are shown in Fig. 3.

The gene 38 protein did not appear to be incorporated into the particles but was later shown to constitute the site at the fiber tip that recognizes the host receptors.

A general feature of tail fiber assembly, as revealed by the SDS gel analysis, was that their translation proceeded whether or not they were assembled into mature fibers and onto the phage. This macromolecular assembly process was not controlled at the level of transcription or translation but entirely by interactions among the precursor proteins. This was a departure from the accepted models of regulation through gene transcription or messenger translation. Since the proximal tip of the proximal half fiber did not bind to the phage baseplate until it was assembled to the distal half fiber, a conformational signal needed to be propagated from the elbow 600A to the proximal tip.

The gene 57 and 38 proteins turned out to be chaperones required for the folding of the gp37 distal half fiber. Thus, in the absence of gp57, the 140,000 gp37 chains accumulated as an insoluble inclusion body state. The high-resolution SDS gels were key to showing that this pelleted material contained the full-length but incorrectly folded gp37 tail fiber chains.

**Figure 4:**
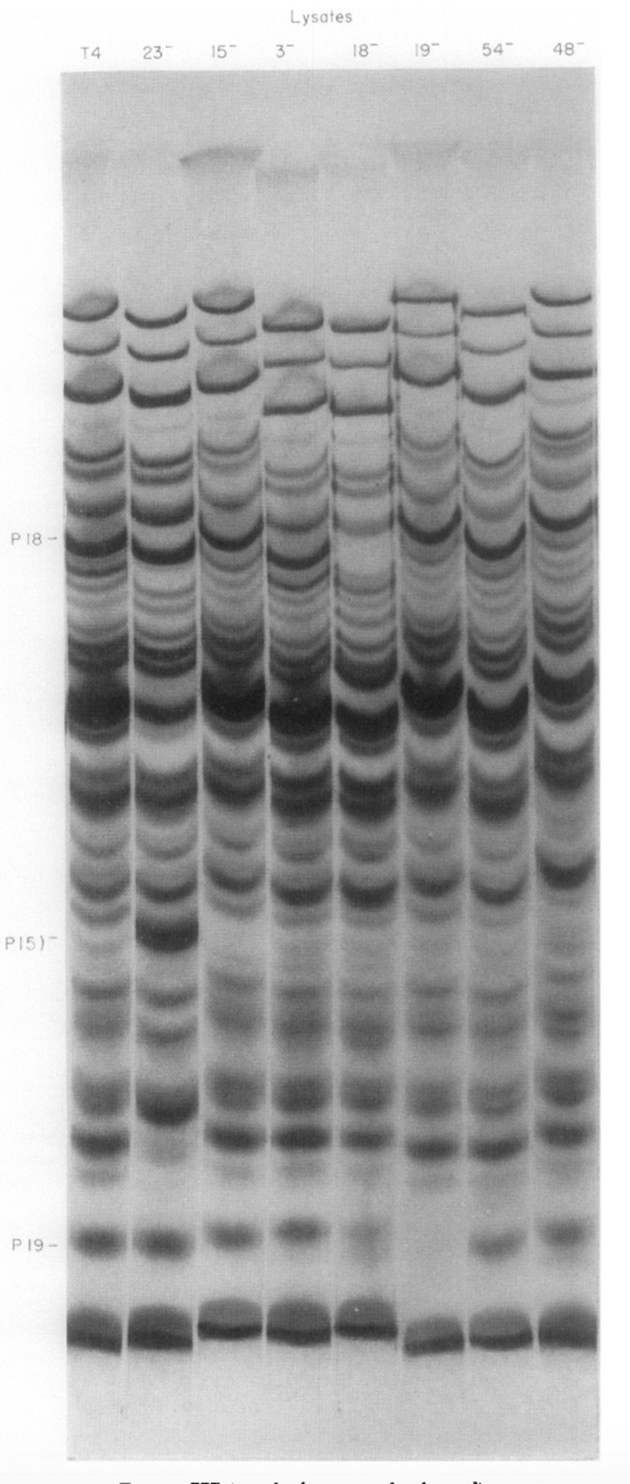
**Threedimensional structure of the C-terminal region of the T4 proximal half fiber** (23, 24)**.** The C-terminal part of the 1289 residue gp34, forming the proximal half of the long tail fibers. The C-terminus forms the elbow joint. The structure of this region of the gp34 trimers (residues 901–1270) is a long triple β-helix that is interspersed with three other domains P3, P4, and P5 that include three anti-parallel β-sheets, one contributed by each of the three monomers.

Though the fibers appear smooth and continuous in negatively stained electron micrographs, the eventual determination of the three dimensional structure by van Raaij and coworkers revealed a more complex morphology (Fig. 4).

## Twenty sequential protein–protein interaction steps in T4 tail assembly

**Figure 5:**
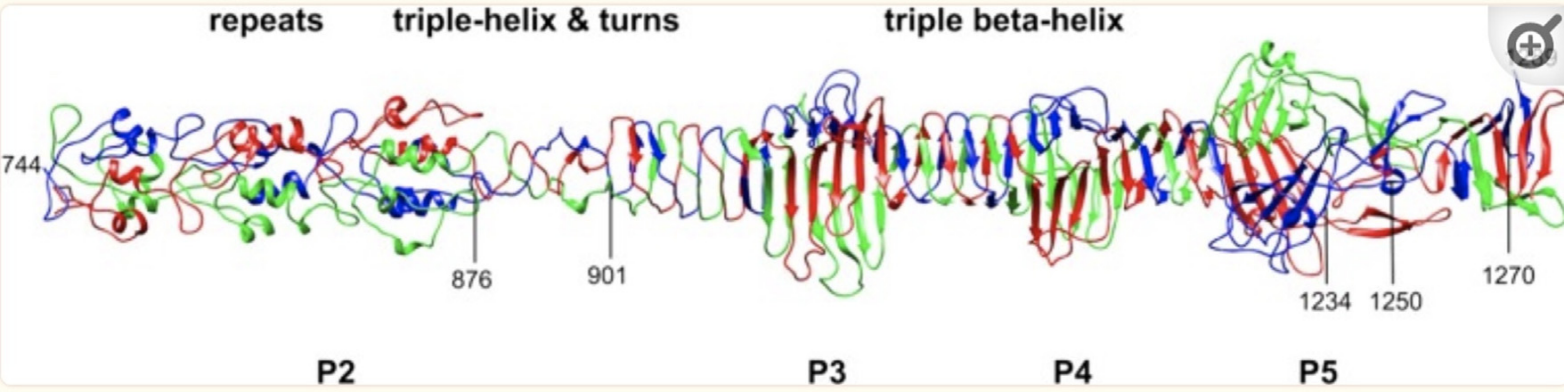
**Identification of the gene 18 tail sheath protein and gene 19 tail tube subunits** (21)**.** Methodology as in ‘Figure 1, but autoradiograms of lysates of cells infected with nonsense mutants in genes controlling tail morphogenesis. The high molecular band missing in the 18- lysate is the major sheath protein, and the lower molecular weight band missing in the next lane over, 19-, is the tail tube subunit. In the absence of the tail tube, the sheath subunits remain unassembled. Note that the rate of translation of the sheath subunit is not affected by whether or not they have been assembled into the tail sheath structure.

With proteins involved in head and tail fiber assembly having been uncovered, the next challenge was to identify the proteins specified by the 20+ genes needed to assemble the baseplate, tail tube, and contractile tail sheath of the phage. I had taken with me the complete collection of amber and temperature-sensitive mutant strains from Caltech to the MRC and then back to MIT. Using Laemmli’s high-resolution SDS gels, Nadia Mykolajewycz and I (25) were able to identify all the polypeptide chains encoded by the “tail” genes (their mutant phenotypes were accumulation of complete heads). Fig. 5 shows SDS-PAGE patterns of the phage structural proteins translated late during infection, with the gp18 sheath protein and gp19 tail tube protein identified as the product of mutant genes.

We then decided to move forward and tackle the steps in the assembly of the very complex baseplate, built as a metastable hexagonal structure. However, upon signals from binding to the cell surface, the baseplate transforms and expands to a six-pointed star which releases the tip of the tail tube to initiate DNA injection into the infected cell (26). The analysis of baseplate assembly was carried out by the very hard-working and extremely careful experimentalist Yoshiko Kikuchi, who joined my lab at MIT as a postdoctoral fellow.

**Figure 6:**
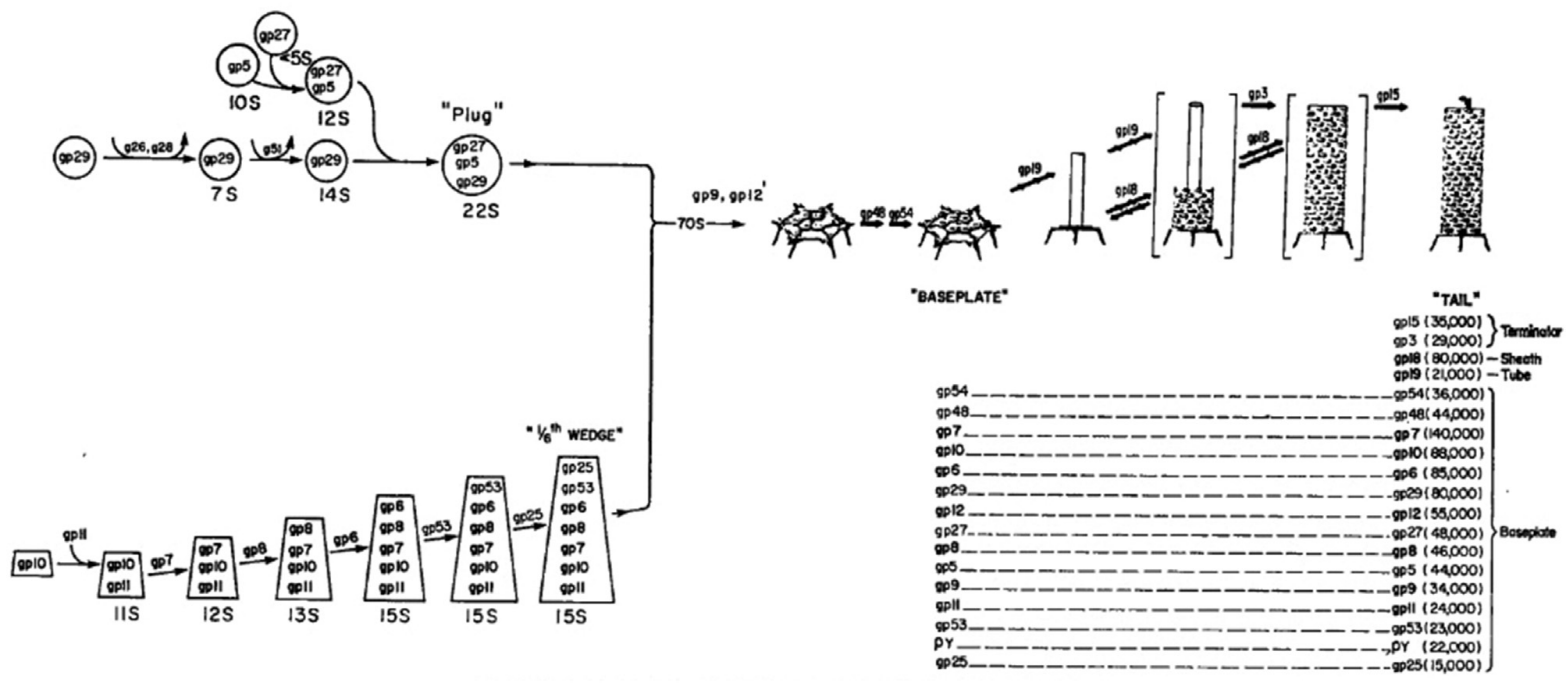
**Pathway for the assembly of the structural proteins participating in T4 baseplate, tail tube, and tail sheath assembly**. One set of proteins assembles into a central hub, which must open during infection to release the tail tube and permit DNA injection. A second set of proteins polymerizes into the outer arms, which then assemble around the central core. Addition of the p48 and p54 proteins activates the baseplate for initiation of tail tube subunit polymerization. If baseplate assembly is blocked by the absence of any protein, the core and tail rube proteins are still translated but do not polymerize into tail tubes or tail sheaths. Those reactions apparently proceed only at the edges of the growing structure, not in solution. Clearly these proteins must be initially translated into a conformation different from the tight binding conformation in the mature structure. That conformational change is what regulates the assembly process and is illustrated more explicitly in Fig. 7 below.

Yoshiko determined the sedimentation behavior of all the unassembled or partially assembled baseplate proteins accumulating in cells missing one of the proteins. This allowed us to determine the baseplate assembly pathway, which had two components: assembly of the central hub and the outer arms (27, 28, 29). The full tail assembly pathway is shown in Fig. 6.

Yoshiko was also able to establish conditions in which these steps proceeded *in vitro*, by refinements of Edgar and Woods’ *in vitro* complementation between extracts made from cells infected with phages harboring mutant genes. This provided confirmation of the model above (Fig. 6).

It should be noted that every one of Yoshiko’s experiments demonstrated that the unassembled proteins required for the later steps in tail assembly, for example the tube and sheath subunits, existed in a soluble state prior to polymerization. These unassembled subunits were biologically competent since mixing extracts of the mutant-infected cells led to the production of infectious T4 phage (27, 28, 29).

**Figure 7:**
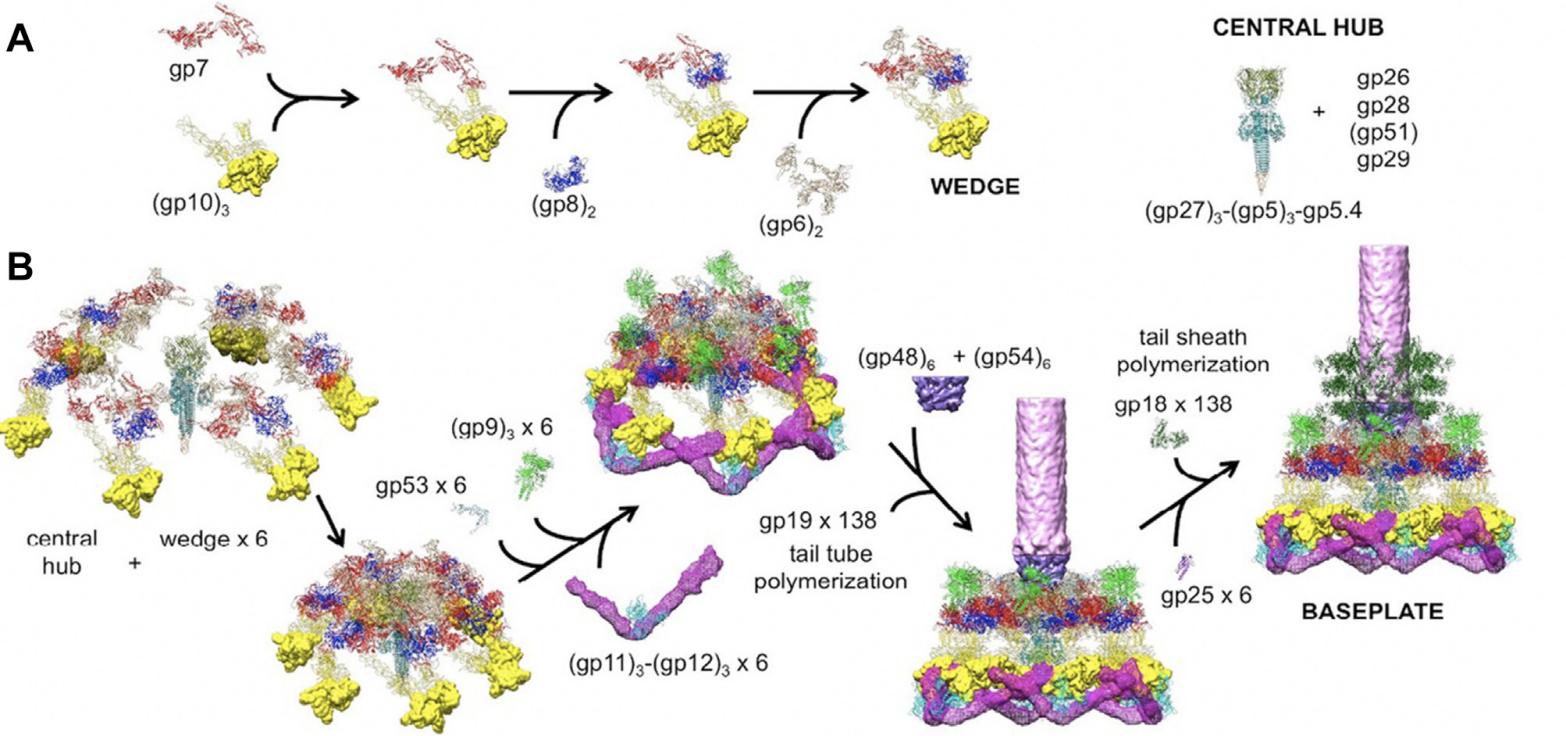
**Three dimensional structures of the proteins involved in baseplate, tail tube, and tail sheath assembly** (30). The baseplate proteins are named for their encoding gene ("gp" = "gene product"). This pathway incorporates cryo-EM and X-ray diffraction data illustrating the morphogenesis of the tail, following the general features shown in Fig. 5.

Though we had already identified the full set of 21 proteins needed to build the phage tail and the complete sequence of their interactions needed to assemble the tail (Fig. 6), 30 more years passed before the structure and organization of these protein components were solved at high resolution by Michael Rossman’s group. Finally Fumio Arisaka, working with Rossman at Purdue, succeeded in determining the 3-D structures of many of the baseplate proteins by cryo-electron microscopy and/or X-ray diffraction (24, 30). The 3-D structures of these proteins are shown in Fig. 7.

**Figure 8:**
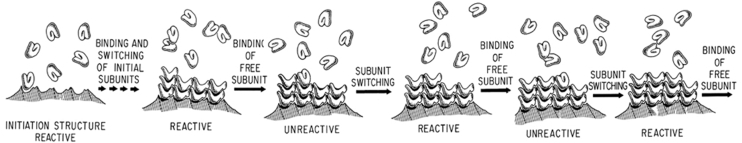
**Control of protein polymerization; conformational activation upon incorporation into the growing structure**. This cartoon of tail sheath polymerization attempts to capture the mechanism of regulation of polymerization, processive conformational change at the growing face of the structure. The conformation reached after release from the ribosome does not associate or polymerize. The polymerization reaction is initiated by binding of the nonreactive subunits to the completed baseplate. This triggers a conformational transition of the bound subunit to an active conformation, which again binds soluble subunits. This reaction propagates until completion of the sheath. In the actual tail assembly pathway, sheath subunits polymerize forming 24 rings of six along the 24 rings of six the tail tube subunits.

It should be noted that the 3-D structures of soluble precursor subunits of the phage tail remain to be determined. We do know that these proteins do not interact until assembly of the baseplate is completed. Then, these tail tube subunits bind to the baseplate to initiate a highly processive tail assembly process, with the requisite protein conformational changes occurring only upon binding to the growing tail structure. This process is illustrated in Fig. 8 for the polymerization of the sheath subunits.

It is worth noting that the enormous amount of 3-D information represented in such studies is generally outside the capacity of students and interested scientific colleagues to assimilate in a normal talk, lecture, or even private reading of the papers. Though I often lectured on phage assembly, I have yet to find a way to ensure that the structural information is assimilated.

## Applications of SDS-PAGE to protein folding, misfolding, and the enigma of inclusion body formation

With the widespread development of gene splicing and cloning technology, biotech and pharmaceutical companies were moving rapidly to produce proteins of therapeutic value in *E. coli*, such as insulin, growth hormones, and many others. To the consternation of the chemical engineers charged with actual production of the protein of interest, recovery of soluble active protein was rare. Far more common was the accumulation of the polypeptide chains coded by the cloned genes, in an aggregated, insoluble, and inactive state as an inclusion body. This was completely mysterious at the time since the predominant view of protein folding was Anfinsen’s hypothesis that correct sequences under native conditions, folded spontaneously to the native state. The cloned sequences were correct, and the cell was certainly a native environment, so the failure of folding was obscure. From my interest in structural proteins, I was familiar with the collagen/gelatin transition and that kinetic factors were often dominant in those reactions. It followed that the aggregated inclusion body state of many cloned proteins was a kinetically trapped off-pathway aggregated state, as is gelatin.

Such kinetically trapped aggregated states were ignored in the biophysical analyses focused on small proteins with folding half times of seconds or fractions of a second. In fact our measurements of the *in vivo* folding and later *in vitro* refolding of a number of phage structural proteins indicated folding half times of many minutes. We also knew that a significant fraction of at least the structural proteins of bacteriophage could end up in such an aggregated state, for example coat proteins in the absence of the GroE chaperonins. In fact, T4 tail fiber chains or Phage P22 tailspike chains carrying temperature-sensitive amino acid substitutions and synthesized at the high restrictive temperature, ended up in such an aggregated state.

**Figure 9:**
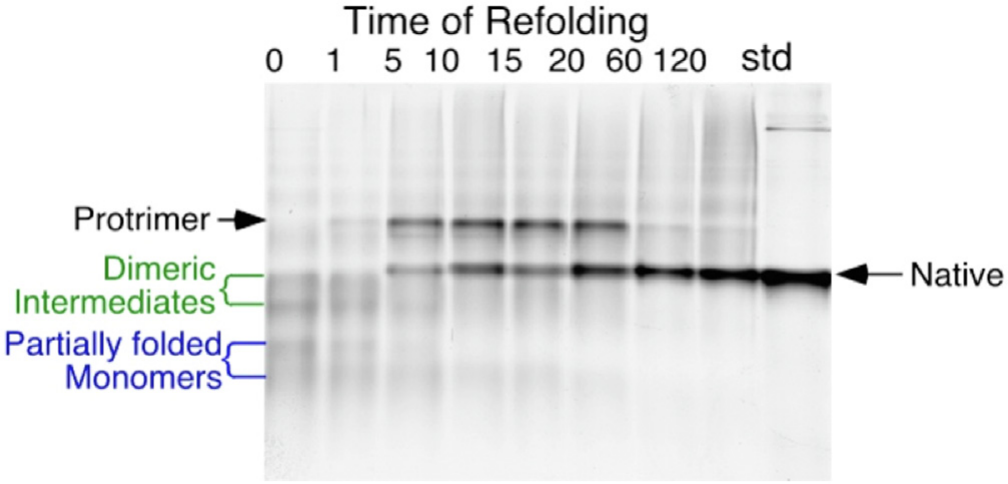
**Direct analysis of *in vitro* folding and misfolding of the tailspike of phage P22 using SDS-PAGE**. Purified P22 tailspikes were denatured with GuHCl *in vitro* and then diluted to buffer to initiate refolding. Samples were electrophoresed in the cold SDS and without prior heating. The partially folded species are sufficiently long lived to be resolved in the gels, where their electrophoretic mobility is determined by the interaction of charge and chain conformation.

The key experiments exploring inclusion body formation were performed with P22-infected *Salmonella* cells. The P22 tailspike turned out to be invaluable for revealing the features of inclusion body formation (31). Robert Seckler and co-workers in Germany had shown that the structure consisted of intertwined B-strands forming a triple B-helix (32). The wrapping of the chains around each other in the triple B-helix mode meant that the single chain folding intermediates could not possibly be in the native state. In fact the pathway included a partially folded trimeric intermediate, the protrimer, whose lifetime was long enough to resolve as a separate band in gels (33, 34). Fig. 9 shows a nondenaturing gel analysis of the *in vitro* folding of the P22 tailspikes, with monomeric, dimeric, and trimeric partially folded intermediates resolved from the native state.

The tailspike, with a melting temperature above 80 °C, was so physiologically stable that there was no possibility the inclusion body state could be derived from denaturation of the native state. Cammie Haase-Pettingel and Anna Mitraki showed this definitively by following the kinetics of inclusion body formation and showing that the aggregated chains derived from intracellular folding intermediates, rather than breakdown of the native state (31, 35).

Though this result did not get much attention from the protein folding or protein biochemistry communities, the results percolated through the Biotech industry. This led to simple physiological approaches to increase yields of native states of recombinant proteins, by, for example, growing cells at the lowest temperature supporting translation of the cloned proteins. Scott Betts showed this cold rescue convincingly for tailspike folding and maturation. Another approach in biotechnology was to purify the very stable inclusion bodies, solubilize the polypeptide chains with a denaturing agent, followed by protein refolding *in vitro*.

**Figure 10:**
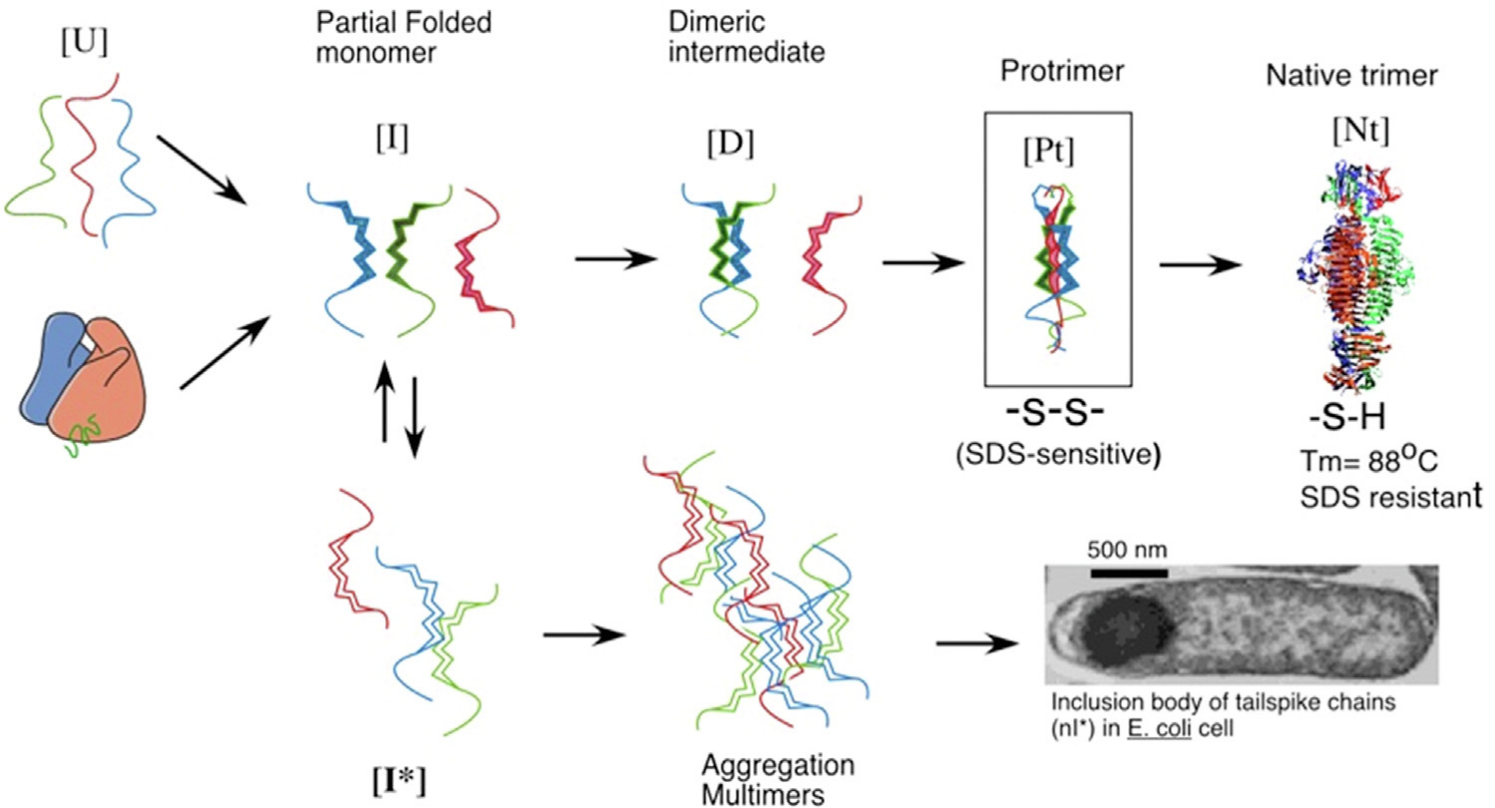
***In vivo* folding, misfolding, and inclusion body formation**. Experiments on the *in vivo* and *in vitro* folding and misfolding of the P22 tailspike are summarized (31, 33, 34, 35). Though the native tailspike is stable to above 80 ^°^C, the early intermediates are thermolabile. This explains the isolation of temperature-sensitive mutations for this thermostable protein—the temperature sensitive stage is in the kinetic folding intermediates, not the native mature trimer. The inclusion body state acts as a kinetic trap for misfolded chains.

These off-pathway misfolding processes were systematically elucidated by the experiments of Margaret Speed, an MIT Chemical Engineering major, working with Professor Danny Wang. Margaret’s very careful characterization of the competition between tailspike refolding and aggregation, both *in vivo* and *in vitro*, led to insights into this problem. Using native gel electrophoresis, she was able to separate the multimeric intermediates along the aggregation pathway from the partially folded intermediates leading to the native state (36, 37). The productive folding and off-pathway interactions for tailspike chains—both *in vitro* and *in vivo*—are summarized in Fig. 10.

While biochemists thought of aggregation as a nonspecific process, bio/pharma production engineers knew that the inclusion body was often a rather pure state of the overexpressed protein. Using mixtures of folding and aggregation intermediates of the P22 coat protein and tailspike, we were able to demonstrate directly that the inclusion body pathways were specific, even when multiple species were aggregating. This explained the well-known experience in biotechnology production that inclusion bodies were concentrated nonnative states of the expressed cloned chains.

## Amino acid sequence control of chain folding

The SDS-PAGE gels permitted assessment of the effects of single amino acid substitutions on the fate of the tailspike chains. The initial analysis showed clearly that the temperature-sensitive mutations originally isolated by R.S. Edgar for structural proteins acted not on the native states but by destabilizing intracellular folding intermediates (31, 33).

**Figure 11:**
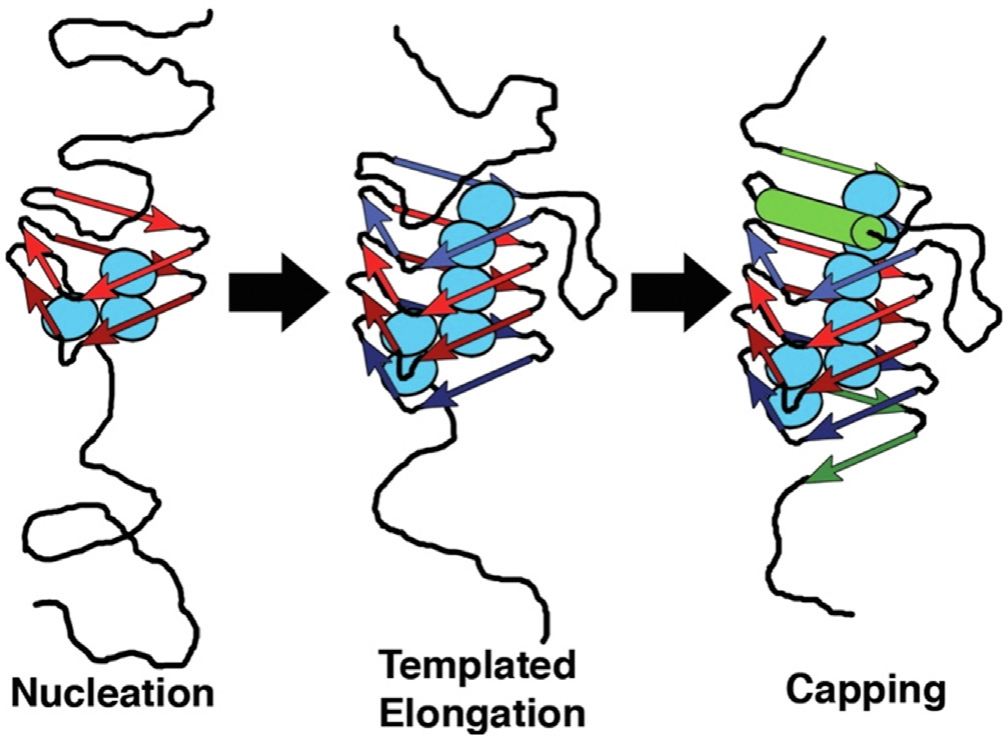
**Control of beta-helix folding by sequential hydrophobic interactions within and between rungs** (38)***.*** In the triple beta-helix fold of the tailspike, hydrophobic residues form long stacks from one rung to the next. The diagrams shows the formation of these processive interactions as organizing the folding of the three chains into the mature highly stable beta-helical fold (38). The diagram attempts to capture initiation, propagation, and termination steps.

Subsequently, we realized that the same methodology could be used to study the behavior of tailspike chains carry absolute—not conditional—lethal amino acid substitutions. These mutant chains never reached the native state but could be tracked with SDS gels. The experiments carried out by Ryan Simkovsky indicated that the key interactions driving the formation of the triple beta helix were hydrophobic interactions in the buried hydrophobic core of the structure (38). These formed long hydrophobic stacks along the length of the triple beta-helix structure. Since these interactions repeated at every rung of the beta helix, they led to a model of sequential buried side-chain interactions driving the beta-helical fold (Fig. 11).

## Further SDS-PAGE developments

The original gels were cast in tubes, which we cracked with a hammer and then sliced the gel lengthwise for drying. Some years later, William Studier described slab gels, which were much more efficient for multiple samples than individual tube gels (39). This method rapidly spread throughout the molecular biology community and has remained the method of choice since then. Coomassie Blue, derived from textile dyes, was already in use for staining proteins. Subsequently the two-dimensional gel was introduced, and of course methods such as Western blotting and Northern blotting depending on the underlying stability of the acrylamide gel separation step.

After the initial brief report in Laemmli’s historic 1970 Nature paper (1), Laemmli and Maizel planned to write an article laying out both the procedures and the theoretical basis of this invaluable technique. Unfortunately, the article was never completed. In 2000, Maizel reviewed the history of the use of SDS in fractionating viral proteins (40), and Aaron Klug published an excellent review of contributions made by him and his co-workers to structural biology (41) but modestly neglected to mention that Laemmli’s SDS-PAGE was developed under his watch.

Laemmli went on to make many significant contributions to our understanding of chromatin dynamics. Maizel continued to elucidate features of animal virus structure and assembly that he had started with the lower resolution SDS gels. I continued to study the genetic control of phage assembly, which led to investigating folding, misfolding, and aggregation of phage structural proteins (Fig. 10). Subsequently my group used our experiences studying off-pathway aggregation to elucidate the mechanism of aggregation of the eye lens crystallin proteins, the root cause of cataracts and a major form of blindness (42, 43).

Though the identity and function of most capsid proteins were sorted out by 1970, their conformation, interactions, and detailed organization awaited the development of high-resolution cryo-electron microscopy over the following three and a half decades, led by Wah Chiu and colleagues (44, 45). Only now are the full mechanistic details emerging of controlling capsid assembly, DNA packaging, and DNA injection (46).

Given the importance of understanding viral particle assembly in this pandemic outbreak, it is important to recognize that high-resolution structure of virions is not sufficient to reveal the critical assembly interactions. The native interactions are docked, stabilized states, but the precursor subunits exist in different conformations. Thus, the coat and scaffolding subunits of the P22 procapsid remain soluble in each other’s absence (47). As with phage tail assembly, the subunits must be conformationally activated by addition to the growing shell.

The bulk of the work Laemmli, Maizel, Klug, and I carried out was funded by public agencies, in the US, UK, and Switzerland, and we never even discussed privatizing the procedures. Both the development of the technique and its rapid propagation throughout the world’s scientific community are testimony to the value of public support for biomedical research and the existence of an open interactive international community. The founders of T4 biology—Max Delbruck, A.H. Hershey, and Salvador Luria—were all active—both pre-WWII and post-WWII—in opposition to Fascism and actively promoted the view that scientists should always share their data, their strains, and their ideas. And of course, we owe Aaron Klug and the MRC a debt of gratitude for providing a supportive, tolerant, and understanding scientific environment.

## Conflict of interest

The author declares no conflicts of interest with the contents of this article
